# Discovery of chemical markers for improving the quality and safety control of *Sinomenium acutum* stem by the simultaneous determination of multiple alkaloids using UHPLC-QQQ-MS/MS

**DOI:** 10.1038/s41598-020-71133-4

**Published:** 2020-08-25

**Authors:** Yu-Feng Huang, Fan He, Can-Jian Wang, Ying Xie, Yan-Yu Zhang, Zhen Sang, Ping Qiu, Pei Luo, Sheng-Yuan Xiao, Jing Li, Fei-Ci Wu, Liang Liu, Hua Zhou

**Affiliations:** 1 Faculty of Chinese Medicine and State Key Laboratory of Quality Research in Chinese Medicine, Macau University of Science and Technology, Taipa, Macao People’s Republic of China; 2 Institute of International Standardization of Traditional Chinese Medicine, Shanghai University of Traditional Chinese Medicine, Shanghai, 201203 People’s Republic of China; 3 Hunan Zhengqing Pharmaceutical Company Group Ltd., Huaihua City, 418000 People’s Republic of China; 4 College of Chinese Medicinal Materials, Jilin Agricultural University, Changchun, 130118 People’s Republic of China; 5 Joint Laboratory for Translational Cancer Research of Chinese Medicine of the Ministry of Education of the People’s Republic of China, Macau University of Science and Technology, Taipa, Macao People’s Republic of China

**Keywords:** Mass spectrometry, Risk factors

## Abstract

*Sinomenium acutum* stem is a popular traditional Chinese medicine used to treat bone and joint diseases. Sinomenine is considered the only chemical marker for the quality control of *S. acutum* stem in mainstream pharmacopeias. However, higenamine in *S. acutum* stem is a novel stimulant that was banned by the World Anti-Doping Agency in 2017. Therefore, enhancing the quality and safety control of *S. acutum* stem to avoid potential safety risks is of utmost importance. In this study, a fast, sensitive, precise, and accurate method for the simultaneous determination of 11 alkaloids in *S. acutum* stem by ultrahigh-performance liquid chromatography coupled with triple quadrupole tandem mass spectrometry (UHPLC-QQQ-MS/MS) was established. This method successfully analyzed thirty-five batches of *S. acutum* stem samples. The average contents of sinomenine, magnoflorine, coclaurine, acutumine, higenamine, sinoacutine, palmatine, magnocurarine, columbamine, 8-oxypalmatine, and jatrorrhizine were 24.9 mg/g, 6.35 mg/g, 435 μg/g, 435 μg/g, 288 μg/g, 44.4 μg/g, 22.5 μg/g, 21.1 μg/g, 15.8 μg/g, 9.30 μg/g, and 8.75 μg/g, respectively. Multivariate analysis, including principal component analysis (PCA), orthogonal partial least square method-discriminant analysis (OPLS-DA), and hierarchical cluster analysis (HCA), were performed to characterize the importance and differences among these alkaloids in *S. acutum* stem samples. As a result, sinomenine, magnoflorine, coclaurine, acutumine, and higenamine are proposed as chemical markers for quality control. Higenamine and coclaurine are also recommended as chemical markers for safety control. This report provides five alkaloids that can be used as chemical markers for improving the quality and safety control of *S. acutum* stem. It also alerts athletes to avoid the risks associated with consuming *S. acutum* stem.

## Introduction

*Sinomenium acutum* stem (Qing Feng Teng in Pinyin) has been a popular Chinese medicine with an extensive and active global market for decades. Alkaloids, dominated by sinomenine, are the principal chemical components of *S. acutum* stem. Pharmacological research and clinical practice have proven the excellent anti-inflammatory and analgesic effects of sinomenine^1^, which is commonly manufactured as a finished product to treat joint diseases and sports injuries^2^. Therefore, sinomenine has been considered a quality marker for this herb in the Chinese Pharmacopoeia, Japanese Pharmacopoeia, Korean Pharmacopoeia, and European Pharmacopoeia^3–6^.

Nevertheless, using only one marker to reflect the real quality of an herbal medicine containing multiple active ingredients has significant limitations. Phytochemical studies have demonstrated that nearly one hundred alkaloids are present in this herb. It possesses types of morphinans, aporphines, protoberberines, benzylisoquinolines and other compounds, such as sinomenine, magnoflorine, coclaurine, acutumine, and higenamine^2,7^. In addition, the safety of the alkaloids in *S. acutum* stem, especially higenamine and coclaurine, should be considered. For instance, since 2017, the World Anti-Doping Agency (WADA) has explicitly banned higenamine as a novel stimulant ingredient^8^. However, higenamine is a natural alkaloid found in several herbs, including *Aconitum japonicum* Thunb., *Tinospora crispa* (L.) Hook.f. & Thomson, *Nandina domestica* Thunb., Gn*etum parvifolium* (Warb.) C.Y. Cheng, and *Asarum heterotropoides* F. Schmidt^9^, as well as in *S. acutum* stem. Coclaurine could also be a potential stimulant because it is a metabolite of higenamine with a similar chemical structure^10–12^. Coclaurine has also been identified in rat urine by gas chromatography/mass spectrometry after higenamine administration^13^.

Moreover, many athletes inadvertently consumed higenamine-containing products and were sanctioned in recent years^14–17^. These accidental doping cases have raised global concern. Higenamine has been detected in sports supplements and herbal products. Some such supplements are even unlabeled or inaccurately labeled in North America and Asia^18^. Athletes may use *S. acutum* stem and related products to treat inflammation and pain, but the health risks of higenamine remain unclear^19^, and little attention is paid to the banned ingredients in this herb. Trace amounts of higenamine and coclaurine can be found in human plasma and urine by extract mass spectrometry after intravenous or oral administration of manufactured herbal products^20,21^. However, the amounts of higenamine and coclaurine in *S. acutum* stem and whether there is a potential safety hazard of using *S. acutum* stem by athletes are still unknown. Therefore, we must study potential doping incidents caused by the unintentional use of herbal products. It is of considerable significance to study whether other alkaloids could be accepted as chemical markers to improve the safety control of *S. acutum* stem.

In this study, a rapid, sensitive, precise and accurate method for the simultaneous quantification of 11 alkaloid compounds in *S. acutum* stem was established for the first time by using ultrahigh-performance liquid chromatography coupled with triple quadrupole tandem mass spectrometry (UHPLC-QQQ-MS/MS). This strategy can be used to strengthen the quality control of *S. acutum* stem, thereby ensuring safety.

## Results

### UHPLC-QQQ-MS/MS optimization

Different solvents and gradient profiles for the mobile phase were compared to achieve a reasonable resolution within 10 min. The separation efficiency and peak symmetry of the analytes were dramatically improved with 0.1% formic acid (A) and acetonitrile (B) as the mobile phase. Because of the wide polarity range of the analytes and the presence of isomers (columbamine and jatrorrhizine), gradient elution was employed and showed a better separation than isocratic elution.

**Figure 1:**
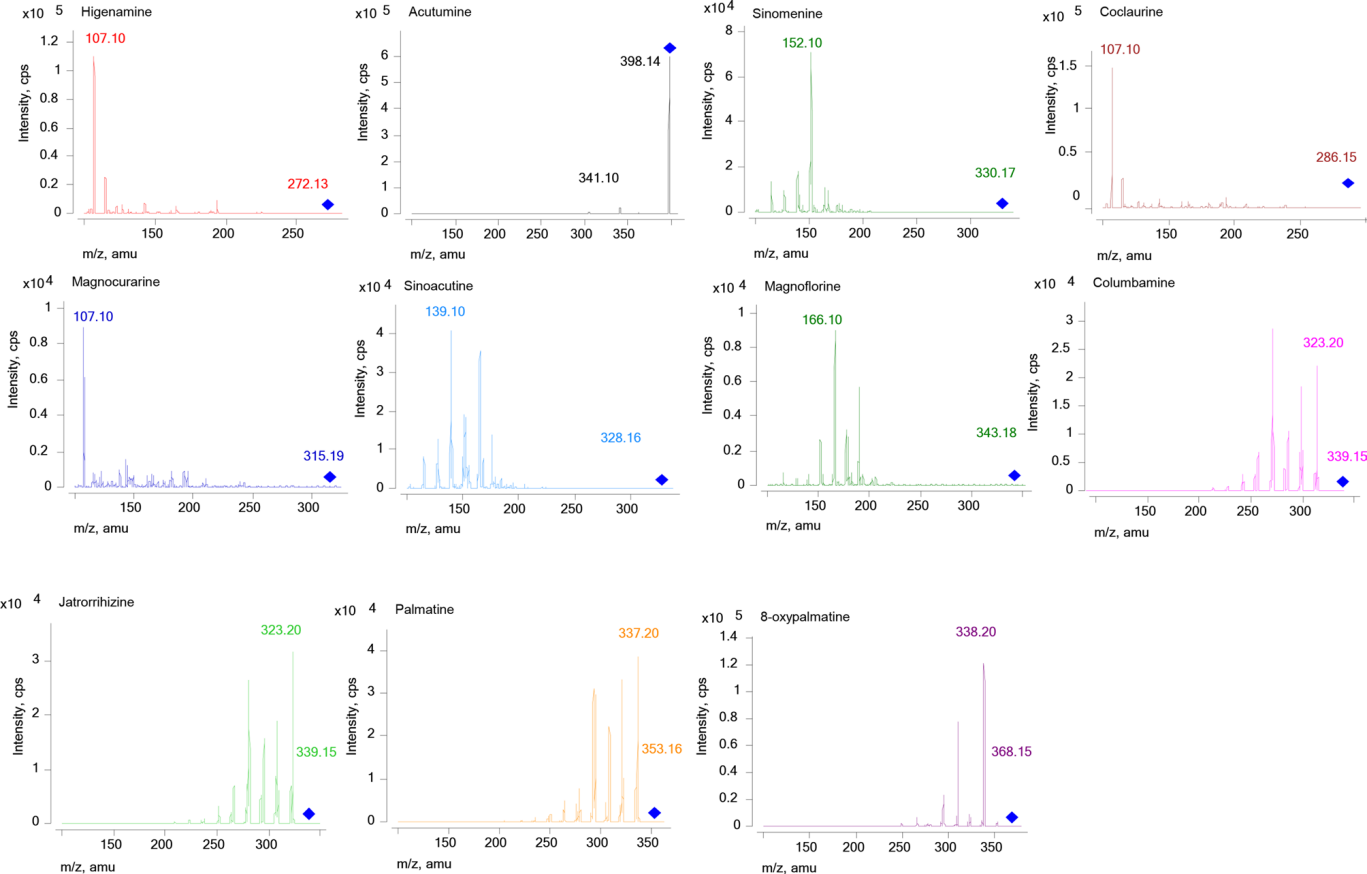
Full scan product ion mass spectra of the 11 alkaloids.

The positive mode provided higher ionization efficiency and sensitivity than negative mode for the MS analysis. The collision energy and fragmentor voltage parameters were optimized to obtain the highest relative abundance of the exclusive ions and product ions in optimized MRM conditions. The final conditions for the collision energy and fragmentor voltage are shown in Table 1. The MS/MS ion spectra of the 11 alkaloids are shown in Fig. 1.

**Table 1:** The transitions, linearity, range, and limits of determination and quantification.

Analytes	Precursor ion (*m/z*)	Product ion (*m/z*)	Fragmentor (eV)	Collision energy (eV)	Calibration curves	Range (ng/mL)	Correlation coefficient (r)	LOD (ng/mL)	LLOQ (ng/mL)
Sinomenine	330.17	152.10	170	76	Y = 7,535.89X + 16,672.91	2.44 × 10^4^–1.56 × 10^5^	0.9997	36.8	55.2
Magnoflorine	343.18	166.10	130	76	Y = 938.59X + 2014.32	990–6.34 × 10^4^	0.9995	264	792
Coclaurine	286.15	107.10	100	24	Y = 113,074.27X + 4,721.15	80.0–4.90 × 10^3^	0.9997	6.12	15.3
Acutumine	398.14	341.10	45	8	Y = 6,754.35X + 1,152.14	120–7.62 × 10^3^	0.9998	58.8	90.5
Higenamine	272.13	107.10	110	24	Y = 64,738.75X + 3,603.34	62.8–4.02 × 10^3^	0.9998	20.1	62.8
Sinoacutine	328.16	139.10	120	96	Y = 22,780.09X − 161.96	20.5–660	0.9998	10.3	20.5
Palmatine	352.16	337.20	170	28	Y = 12,639.43X − 47.42	17.1–550	0.9995	8.53	17.1
Magnocurarine	315.19	107.10	110	36	Y = 6,164.51X − 17.48	45.2–720	0.9995	11.3	45.2
Columbamine	339.15	323.20	170	28	Y = 16,054.15X − 92.23	27.3–440	0.9995	13.6	27.3
8-Oxypalmatine	368.15	338.20	110	28	Y = 46,182.03X + 72.94	10.7–340	0.9998	5.34	10.7
Jatrorrhizine	339.15	323.20	140	28	Y = 17,136.94X − 32.96	7.72–250	0.9996	3.86	7.72

### Extraction method optimization

**Figure 2:**
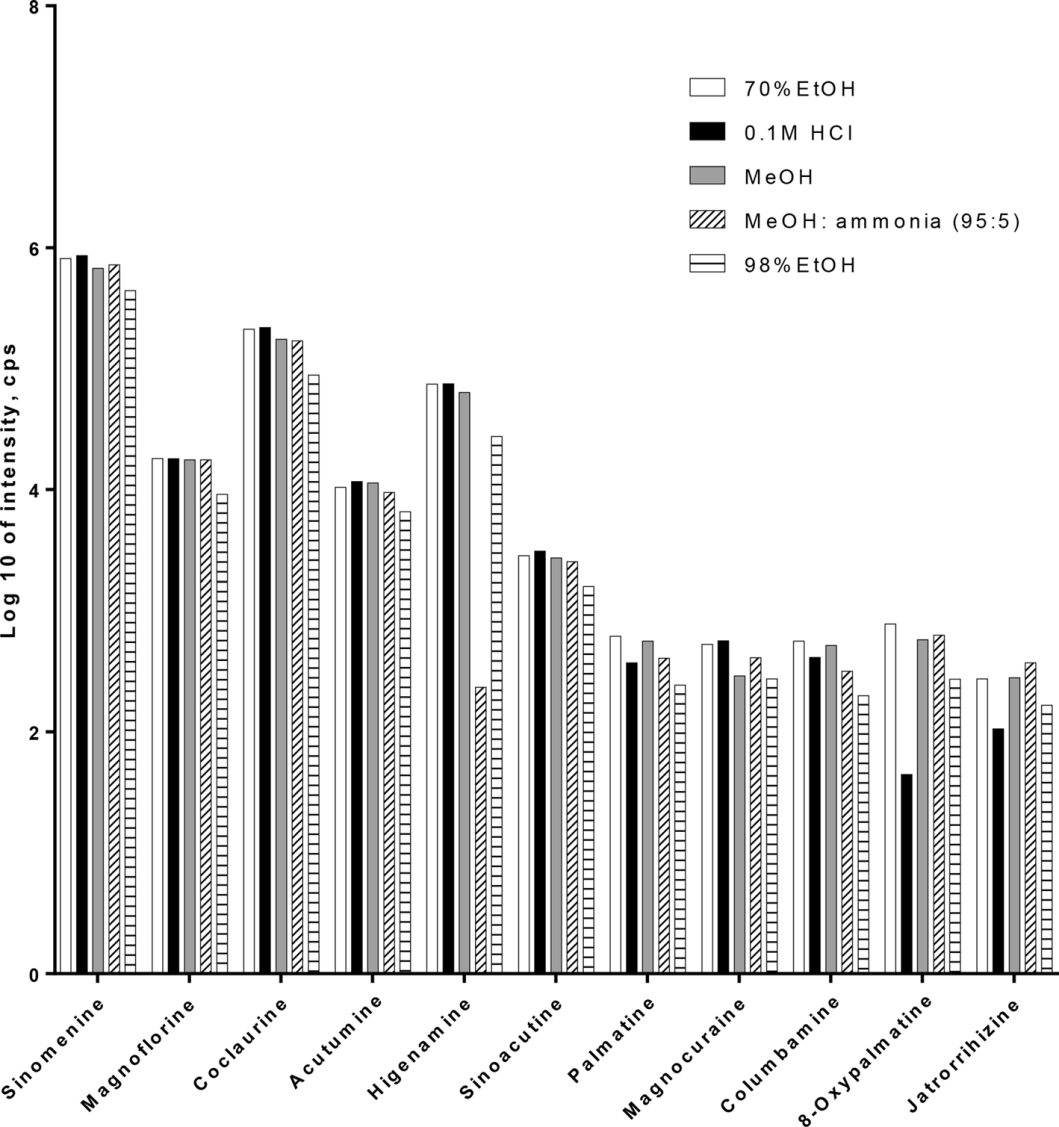
Optimization of the extraction of 11 alkaloids from *Sinomenium acutum* stem sample (Q5) by different solvents.

Since the contents of these 11 compounds are significantly different, the sample extraction method was optimized to achieve efficient extraction for all the compounds based on the Chinese Pharmacopeia method (2015 edition), and five extraction solvents, i.e., 20 mL of 70% ethanol solution, 20 mL of 98% ethanol solution, 20 mL of 0.1 M hydrochloric acid solution, 20 mL of methanol solution and 20 mL of methanol with ammonia (V/V 95:5) solution, were compared. Based on the results (Fig. 2), 20 mL of 70% ethanol solution was selected to efficiently extract the 11 alkaloids from the *S. acutum* stem.

### Method validation

The regression equations, linear ranges, correlation coefficients, limits of detection (LODs), and lower limits of quantification (LLOQs) of the 11 compounds are listed in Table 1. All the calibration curves exhibited excellent linearity with correlation coefficients (R values) in the range of 0.9995–0.9998. The LOD was set to a signal-to-noise ratio (S/N) not less than three, and the LLOQ was not less than approximately ten.

The precision was determined by continuously testing one concentration of the mixed standard solution six times. Sample Q29 was separated into six portions, and each portion was extracted separately to evaluate the repeatability of this method. The test solution of sample Q29 was employed to evaluate the stability over 0, 2, 4, 6, 8, and 12 h in one day at room temperature. The results for all the compounds, which are summarized in Table 2, indicate that the instrument has good precision, the method is repeatable, and the compounds in the sample solution are sufficiently stable for accurate and precise analysis within 12 h at room temperature (Supplementary information).

**Table 2:** The precision, repeatability, stability, and recovery of 11 alkaloid compounds.

Analytes	Precision (RSD%, n = 6)	Repeatability (RSD%, n = 6)	Stability (RSD%, n = 6)	Recovery (n = 6)				
				Original (μg)	Spiked (μg)	Detected (μg)	Recovery (%)	RSD (%)
Sinomenine	2.28	1.50	0.89	5.94 × 10^3^	5.89 × 10^3^	1.18 × 10^3^	99.4	2.37
Magnoflorine	1.28	2.26	0.63	1.98 × 10^3^	1.58 × 10^3^	3.62 × 10^3^	103.5	1.07
Coclaurine	1.46	1.15	2.01	126	147	271	98.8	3.52
Acutumine	0.72	2.25	3.67	83.8	114	200	101.7	1.88
Higenamine	1.01	1.35	3.26	86.5	80.4	166	99.1	2.24
Sinoacutine	1.00	4.53	2.17	8.98	9.84	18.8	99.6	3.52
Palmatine	2.60	4.00	1.55	10.3	10.8	20.9	97.7	3.72
Magnocurarine	2.06	4.54	1.95	5.12	7.23	12.4	100.0	3.67
Columbamine	3.44	3.83	1.90	8.50	8.74	17.2	99.9	2.04
8-Oxypalmatine	3.03	4.63	4.47	1.16	1.37	2.55	101.5	1.84
Jatrorrhizine	2.19	4.15	4.58	3.53	3.71	7.24	100.1	2.59

Six portions (approximately 0.25 g each) of sample Q29 were accurately weighed and extracted separately for the recovery test. The results (Table 2) show that the recovery rates of all 11 alkaloids were within the range of 97.7–103.5% with RSDs not more than 5%, indicating that this method is accurate enough to measure the contents of these 11 compounds in *S. acutum* stem.

### Sample determination

**Figure 3:**
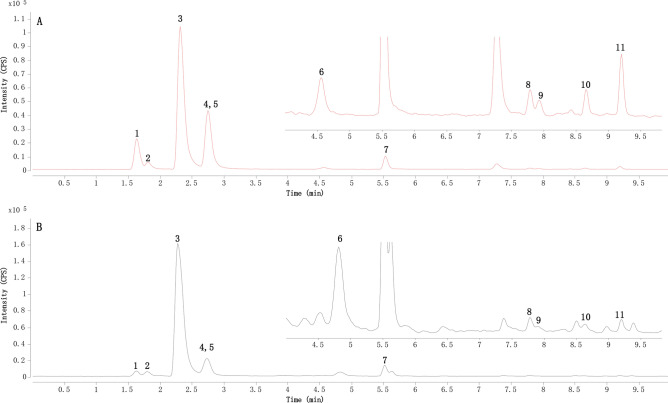
Representative multiple reaction monitoring (MRM) chromatograms of the mixed standard solution (**A**) and test solution of *Sinomenium acutum* stem sample (Q5) (**B**); 1. higenamine; 2. acutumine, 3. sinomenine; 4. coclaurine, 5. magnocurarine, 6. sinoacutine, 7. magnoflorine, 8. columbamine, 9. jatrorrhizine, 10. palmatine, and 11. 8-oxypalmatine.

Thirty-five batches of samples were tested in duplicate by the established method. The representative MRM chromatograms of the reference standard mixture and sample Q5 are shown in Fig. 3. The content ranges of sinomenine, magnoflorine, coclaurine, acutumine, higenamine, sinoacutine, palmatine, magnocurarine, columbamine, 8-oxypalmatine, and jatrorrhizine from the 35 batches of *S. acutum* stem were 14.6–31.5 mg/g, 3.35–9.72 mg/g, 182–934 μg/g, 98.3–1.13 × 10^3^ μg/g, 29.0–794 μg/g, 14.9–86.3 μg/g, 5.40–104 μg/g, 11.5–36.2 μg/g, 4.92–33.7 μg/g, 1.02–50.0 μg/g, and 2.53–26.5 μg/g, respectively. The average contents of these alkaloids were 24.9 ± 4.76 mg/g, 6.35 ± 1.46 mg/g, 435 ± 161 μg/g, 435 ± 213 μg/g, 288 ± 181 μg/g, 44.4 ± 14.9 μg/g, 22.5 ± 20.4 μg/g, 21.1 ± 5.91 μg/g, 15.8 ± 5.80 μg/g, 9.30 ± 11.3 μg/g, and 8.75 ± 4.00 μg/g, respectively.

### Multivariate analysis

**Figure 4:**
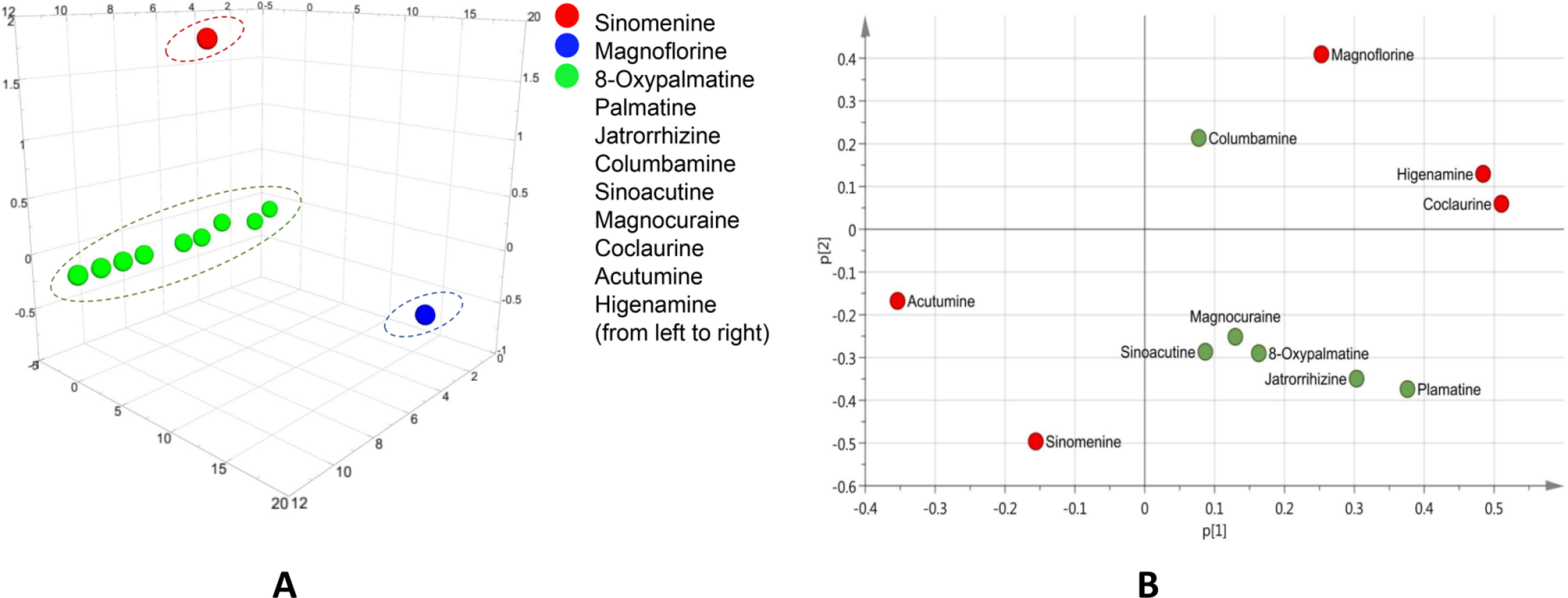
3D score scatter plot (**A**) and loading scatter plot (**B**) of principal component analysis (PCA) showing the contents of 11 alkaloids in the 35 batches of *Sinomenium acutum* stem samples.

**Figure 5:**
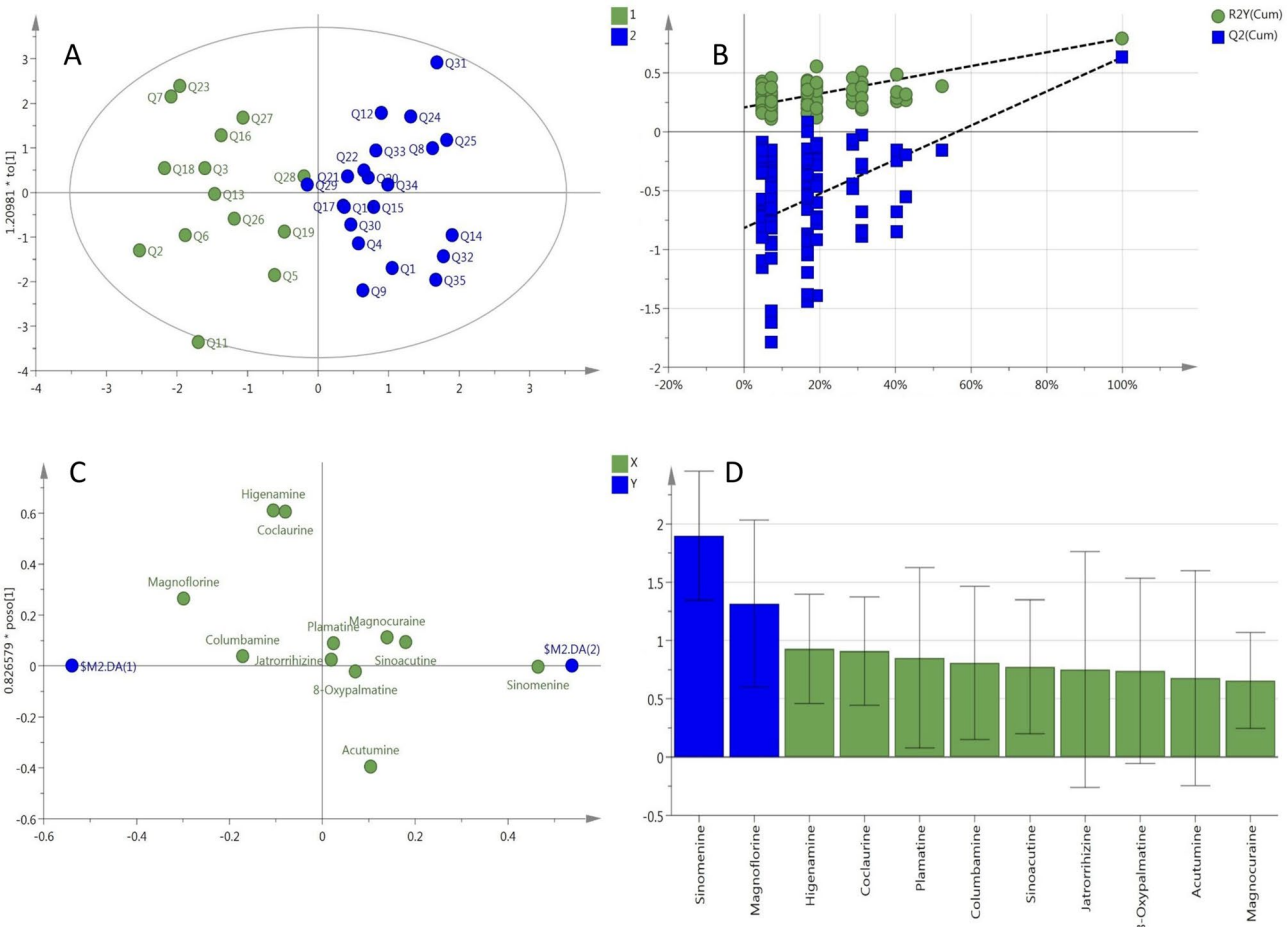
OPLS-DA of 35 batches of *Sinomenium acutum* stem samples. (**A**) Score scatter plot of *S. acutum* stem from the high-content group and low-content group; (**B**) plot of the permutation test; (**C**) loading scatter plot of variables; and (**D**) plot of the variable importance for the projection (VIP).

Principal component analysis (PCA) analysis showed that the cumulative contribution of the first three principal components was 64.3%. The 3D score scatter plot (Fig. 4A) showed that the 11 alkaloids could be divided into three groups, i.e., sinomenine in Group 1, magnoflorine in Group 2, and all the others in Group 3. The orthogonal partial least square method-discriminant analysis (OPLS-DA) model resulted in a (1 + 3) component with the variables of R2 (X) of 0.614, R2 (Y) of 0.792, and Q2 of 0.633. As shown in Fig. 5A, the samples were differentiated into two groups by the OPLS-DA model. The low content group is on the left side of the score plot, while the high content group is on the right side. In the loading plot (Fig. 5C), sinomenine, magnoflorine, higenamine, coclaurine, and acutumine were further from the origin. The plot in Fig. 5D displays the variable importance for the projection values (VIPs) of all variables. The VIPs of OPLS-DA demonstrated that sinomenine and magnoflorine had the greatest influence.

**Figure 6:**
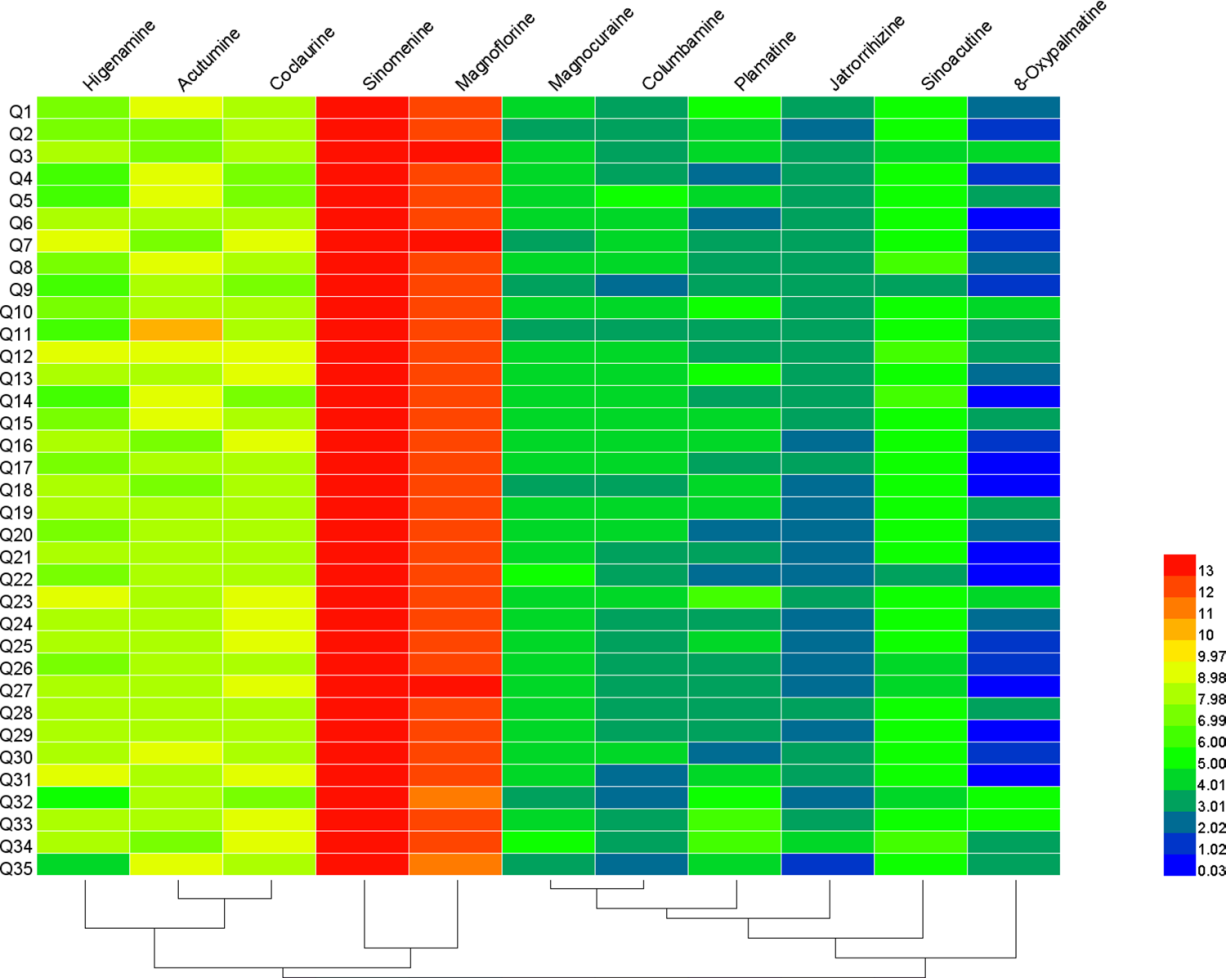
Dendrogram and heatmap of hierarchical cluster analysis (HCA) showing the contents of 11 alkaloids in the 35 batches of *Sinomenium acutum* stem samples. The red color corresponds to a higher concentration, while the blue color corresponds to a lower concentration.

Hierarchical cluster analysis (HCA) showed that the 11 alkaloids could be divided into three groups, as shown in Fig. 6. Sinomenine and magnoflorine were present at high levels. Higenamine, acutumine, and coclaurine were present at moderate levels. Magnocurarine, sinoacutine, columbamine, jatrorrhizine, palmatine, and 8-oxypalmatine were present at low levels.

## Discussion

In this study, thirty-five batches of *S. acutum* stem samples were acquired from local hospitals or pharmacies in China and were determined by the optimized UHPLC-QQQ-MS/MS method. Hua Zhou authenticated all the samples, and the voucher specimens are stored at the State Key Laboratory of Quality Research in Chinese Medicine (Macau University of Science and Technology). The sample number, sample origin, and collection location for each sample are shown in Table 3, and the samples are highly representative of this herb.

**Table 3:** The information of the collected *Sinomenium acutum* stem samples.

Sample number	Sample origin	Collection locations
Q1	Jiangsu Province	Shanghai
Q2	Jiangsu Province	Shanghai
Q3	Unknown	Inner Mongolia Autonomous Region
Q4	Unknown	Shanghai
Q5	Unknown	Shanghai
Q6	Sichuan Province	Nanning, Guangxi Autonomous Region
Q7	Unknown	Shanghai
Q8	Huaihua, Hunan Province	Hubei Province
Q9	Sichuan Province	Linyi, Shandong Province
Q10	Sichuan Province	Shandong Province
Q11	Hubei Province	Shanghai
Q12	Hubei Province	Shanghai
Q13	Hubei Province	Shanghai
Q14	Hubei Province	Shanghai
Q15	Hubei Province	Shanghai
Q16	Anhui Province	Shanghai
Q17	Hubei Province	Shanghai
Q18	Anhui Province	Shanghai
Q19	Hubei Province	Shanghai
Q20	Unknown	Changchun, Jilin Province
Q21	Hubei Province	Shanghai
Q22	Hunan Province	Guangdong Province
Q23	Unknown	Guangzhou, Guangdong Province
Q24	Jiangsu Province	Shanghai
Q25	Hubei Province	Dalian, Liaoning Province
Q26	Jiangsu Province	Shenyang, Liaoning Province
Q27	Anhui Province	Bozhou, Anhui Province
Q28	Anhui Province	Bozhou, Anhui Province
Q29	Hubei Province	Zhuhai, Guangdong Province
Q30	Anhui Province	Beijing
Q31	Zhunyi, Guizhou Province	Zhunyi, Guizhou Province
Q32	Xinyang, Henan Province	Xinyang, Henan Province
Q33	Jinggangshan, Jiangxi Province	Jinggangshan, Jiangxi Province
Q34	Baoji, Shanxi Province	Baoji, Shanxi Province
Q35	Baoji, Shanxi Province	Baoji, Shanxi Province

**Figure 7:**
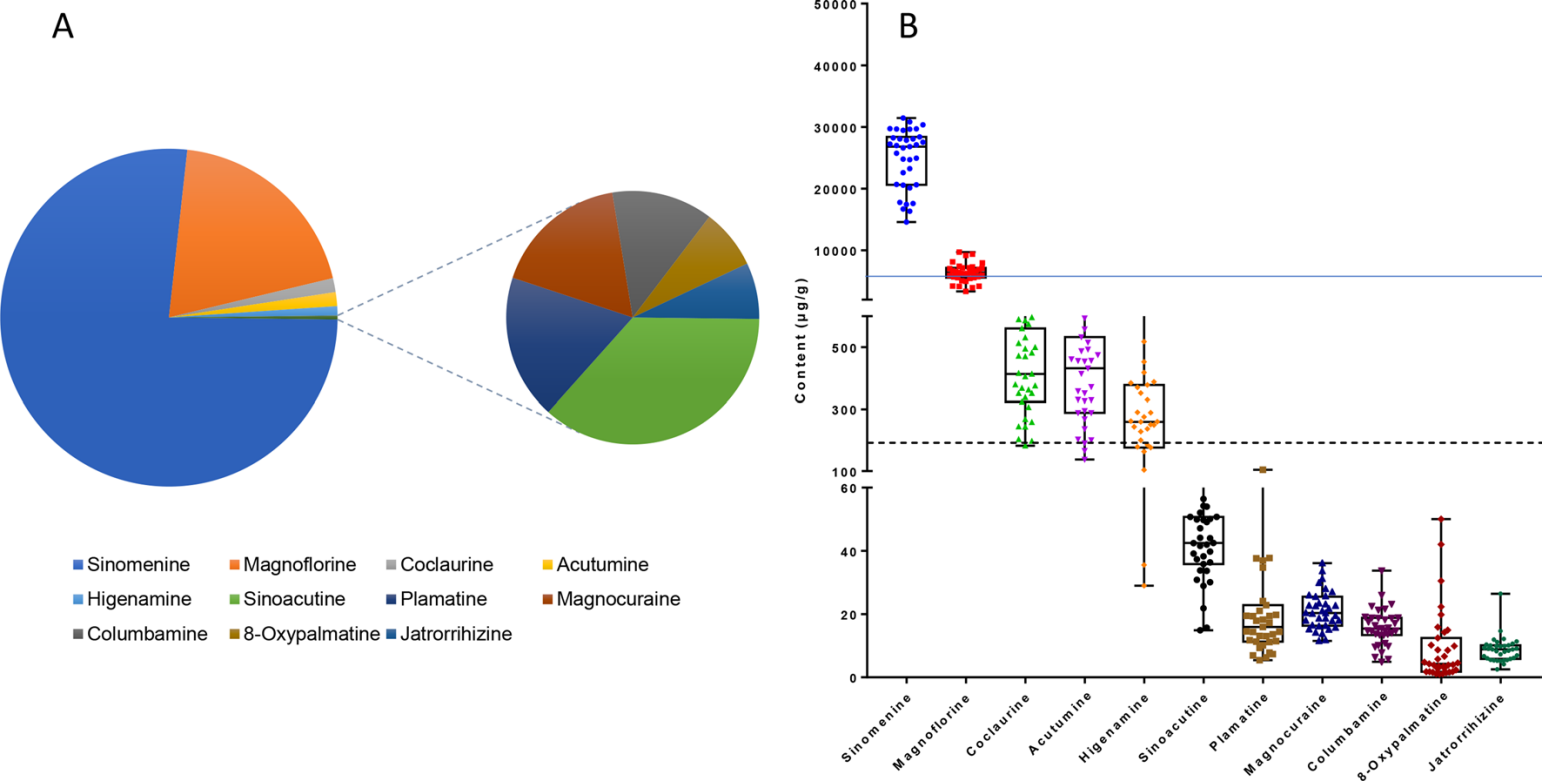
The contents of 11 alkaloids in the 35 batches of *Sinomenium acutum* stem samples. (**A**) The parent composite chart shows the content ratio of each component. (**B**) Blue solid line: the lower limit of the content of sinomenine in *S. acutum* stems according to the Chinese Pharmacopeia and the European Pharmacopeia (not less than 0.5%). Black dotted line: the lower limit of compounds that should be quantitatively controlled according to the Chinese Pharmacopeia (not less than 0.02%).

The average total content of these 11 alkaloids was 32.6 ± 7.17 mg/g. These alkaloids can be empirically divided into three groups (Fig. 7) based on their abundance. Sinomenine and magnoflorine, which are present at the milligram level and account for 76.6 and 19.5%, respectively, of total content, can be classified as high-abundance compounds. Coclaurine, acutumine, and higenamine, which are present at the microgram level and account for 1.34, 1.34, and 0.885%, respectively, of the total content, can be classified as moderate-abundance compounds. Moreover, the remaining six alkaloids, sinoacutine, palmatine, magnocurarine, columbamine, 8-oxypalmatine, and jatrorrhizine, although at the microgram level, account for less than 0.374% of the total content and therefore can be classified as low-abundance compounds. Magnoflorine is a homolog of sinomenine, and they have similar phenanthrene structures and similar biosynthetic pathways^22^, which could be the reason that they are in the same group. A similar explanation can be applied in the case of higenamine and coclaurine^11^.

PCA can simplify complex information by replacing the original variable index with a small number of comprehensive indicators utilizing dimension reduction. In the present study, PCA was used to analyze the distribution pattern of the 11 alkaloids in the *S. acutum* stem. The result of the cumulative contributions meant that the original information of the dataset was basically retained. The 2D loading scatter plot (Fig. 4B), which provided useful information to identify the essential features in the PC1 and PC2 dimensions, showed that sinomenine, magnoflorine, coclaurine, acutumine, and higenamine were located at the edges of the axes, demonstrating the greater correlations with PC1 and PC2 and implying that these five compounds were essential.

OPLS-DA is a supervised discrimination method. Figure 5B shows the modeling used to predict the specific components with the most significant influence on the samples. It demonstrated the considerable quality of the model. Samples with more substantial VIP (> 1.0) are generally more related to sample classification. We found that the VIP values of sinomenine and magnoflorine were more than 1.0, and those of higenamine and coclaurine were very close to 1, and these compounds were in the top four. Therefore, as indicated by the plots in Fig. 5C,D, sinomenine, magnoflorine, higenamine, and coclaurine played an essential role in the *S. acutum* stem samples.

The HCA results were also consistent with the results of content determination, PCA, and OPLS-DA above. Therefore, multivariate analysis verified that sinomenine, magnoflorine, higenamine, coclaurine, and acutumine were the main chemical components of the *S. acutum* stem.

According to the Chinese Pharmacopeia, the following three requirements should be used for the selection of potential chemical markers. The content of the marker in the medicinal material should be higher than 0.02%. The corresponding specific or active ingredients selected as markers for content determination should be involved in the function or bioactivity of the Chinese medicines. A multicomponent detection method should be used when a single component cannot reflect the medicinal materials' overall activity^23^. The pharmacological activities of sinomenine^2,24–26^, magnoflorine^14,27^, coclaurine^28^, acutumine^29^, and higenamine^30,31^ well represent the main indications or bioactivities of *S. acutum* stem, which are anti-inflammation, analgesia, anti-hypertension, anti-arrhythmia, anticancer, and immunomodulation^32^. Therefore, based on the multivariate analysis results and the bioactivities of these components, it was reasonable and representative to identify these specific alkaloids, i.e., sinomenine, magnoflorine, higenamine, acutumine, and coclaurine, as chemical markers of *S. acutum* stem. These alkaloids are recommended for improving the quality control of *S. acutum* stem in pharmacopeias and for use as relevant standards in the future. The use of sinomenine and magnoflorine as chemical markers was also consistent with the published literature conclusions^2,33^. The other three alkaloids proposed together with sinomenine and magnoflorine better reflect the overall quality of this herb.

For the safety control of *S. acutum* stem, higenamine and coclaurine are proposed as chemical markers. Before discussing the safety, we determined that the sinomenine content in each of the samples tested in this study met the requirements of the pharmacopoeias^3,4^. These samples were qualified under the existing standards.

In this study, higenamine and coclaurine were found in *S. acutum* stem at high levels. Therefore, athletes should consider this herbal medicine and its products with caution. The WADA stipulates that all selective and nonselective beta-2 agonists, including all optical isomers, are prohibited. Higenamine is a β-androgenic receptor agonist. It possesses lipolytic activity and can improve cardiac left ventricular function. Therefore, it can promote the growth of skeletal muscle. This is the main reason that higenamine is explicitly banned as a novel stimulant ingredient^8^.

The average contents of higenamine and coclaurine in the *S. acutum* stem were 288 μg/g and 435 μg/g, respectively, in the present study. The recommended oral dosage of *S. acutum* stem is 6–12 g per day according to the Chinese Pharmacopoeia. A previous study reported that the maximum urinary concentration of higenamine in humans was 0.2–0.4 ng/mL within 10–12 h after oral administration of the herbal product containing 19.8 μg higenamine, and the maximum urinary concentration of coclaurine was 0.3–1.0 ng/mL (corresponding to 4.5 μg of coclaurine)^21^. Therefore, we initially speculated that the maximum urinary concentration ranges of higenamine and coclaurine that could be detected after oral *S. acutum* stem treatment for 10–12 h were 17.5–69.9 ng/mL and 39.5–264 ng/mL, respectively, if the ingredients were completely extracted. The anti-doping organization required that the concentration of higenamine in urine should not be more than 10 ng/mL^34^. Apparently, when athletes take regular doses of *S. acutum* stem, the urinary concentration of higenamine exceeds the stimulant detection threshold. Even worse, because of the enzymatic conversion between coclaurine and higenamine, the total concentration of stimulants may also increase in the human body. Therefore, according to the existing standards, these qualified herbs still pose a notable safety risk. With the widespread use of *S. acutum* stem and its products, there is a high risk that unintentional higenamine doping could be detected. This situation also reminds industry and regulatory bodies that higenamine and coclaurine can be used as chemical markers for the safety control for *S. acutum* stems. It is better to warn athletes to use caution on the packaging of related products. This study also alerts athletes that they should be very cautious when they are seeking assistance from herbal medicines to relieve inflammation and pain caused by sports injuries.

## Materials and methods

### Reagents and chemicals

**Figure 8:**
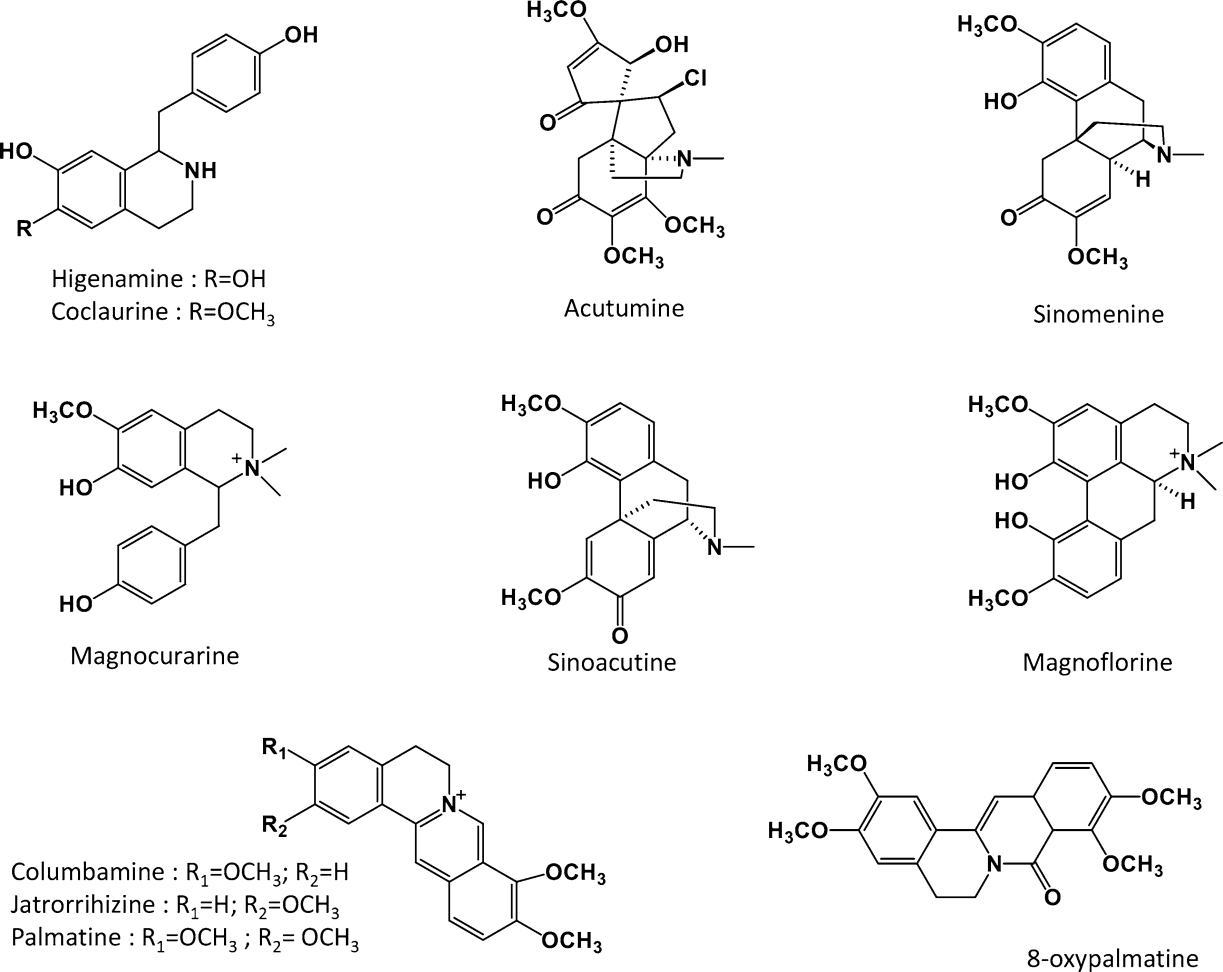
The chemical structures of the 11 alkaloids.

Sinomenine, magnoflorine, sinoacutine, columbamine, and coclaurine as chemical reference standards were purchased from Chengdu Chroma Biotechnology Co., Ltd. (Chengdu, P.R. China). Higenamine, acutumine, magnocurarine, jatrorrhizine, palmatine, and 8-oxypalmatine as reference standards were purchased from Shanghai Chenyi Biotechnology Co., Ltd. (Shanghai, P.R. China). All compounds were at a purity of ≥ 98.0%, and the chemical structures are given in Fig. 8. Formic acid from Sigma-Aldrich Corporation (St. Louis, MO, USA), acetonitrile from Anaoua Chemicals Supply (Houston, TX, USA), and methanol from Anaoua Chemicals Supply (Cleveland, OH, USA) were of HPLC grade. Ethyl alcohol from Anaoua Chemicals Supply (Cleveland, OH, USA) and other chemicals and reagents were of analytical grade.

### Instrument and analytical conditions

An ultrasonic cleaner (i-Quip, Shanghai, China) was used to extract the alkaloids from the samples. A Milli-Q ultrapurification system (Millipore, Bedford, MA, USA) was used to produce ultrapure water. A UHPLC system (1290 series, Agilent Technologies, Santa Clara, CA, USA) coupled with a QQQ-MS/MS (6460 series, Agilent Technologies, Santa Clara, CA, USA) was used to quantitatively detect the 11 alkaloids.

Chromatographic separation was performed on a Waters ACQUITY UPLC HSS C18 SB column (2.1 mm × 100 mm, 1.7 μm, Waters, Milford, MA, USA) at 30 °C with a mobile phase consisting of 0.1% formic acid (A) and acetonitrile (B) in the following gradient: 0–4 min, 16–16% B; 4.01–7 min, 30–30% B; 7.01–10 min, 70–70% B. The injection volume was 2 μL, and the flow rate was 0.35 mL/min. Mass Hunter software (Agilent Technologies, Santa Clara, CA, USA) was used for optimization and quantification. The 11 alkaloids were detected using multiple reaction monitoring (MRM) and an electrospray ionization (ESI) source in positive ion mode. The transitions of the 11 compounds are shown in Table 2. The other parameters were as follows: drying gas (N_2_) flow rate, 11.0 L/min; drying gas temperature, 300 °C; nebulizer, 15 psig; and capillary voltage, 4,000 V.

### Standard solution preparation

**Figure 9:**
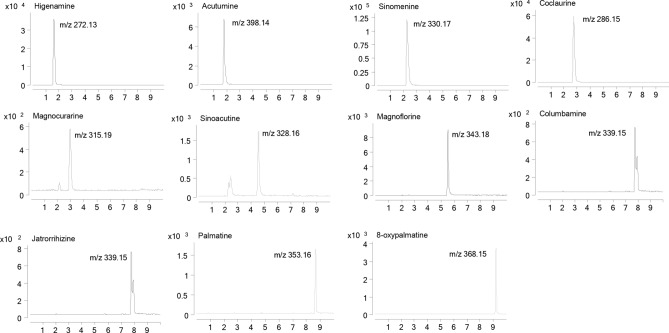
The extracted ion chromatograms (EICs) of the 11 alkaloids.

Appropriate amounts of the reference standards of the 11 alkaloids were dissolved in 70% ethanol to prepare stock solutions. The mixed standard solution was obtained by accurately mixing the 11 stock solutions and diluting them with 70% ethanol. The final concentrations of sinomenine, magnoflorine, coclaurine, acutumine, higenamine, sinoacutine, palmatine, magnocurarine, columbamine, 8-oxypalmatine and jatrorrhizine in the mixed solution were 156, 63.4, 4.90, 7.62, 4.02, 0.656, 0.546, 0.723, 0.437, 0.342 and 0.247 μg/mL, respectively. Two microliters of the mixed standard solution was injected into the UHPLC-QQQ-MS/MS system, and the extracted ion chromatogram (EIC) of the 11 reference standards is shown in Fig. 9. All the standard solutions were stored at 4 °C until use.

### Method validation and test solution preparation

The method was validated in Supplementary information for linearity, precision, repeatability, stability, and recovery following the Guidelines for the Validation of Quality Standard of Traditional Chinese Medicine (Chinese Pharmacopoeia, 2015 Edition, Volume 1) and the United States Food and Drug Administration Bioanalytical Method Validation (US Food and Drug Administration, 2001).

The sample powder (0.5 g passed through a 50 mesh sieve) was accurately weighed in a conical flask with a stopper. The flask was supplemented with 20.0 mL of 70% ethanol, well stoppered, and accurately weighed. Next, the flask was ultrasonicated for 20 min (power 150 W, frequency 20 kHz), cooled to room temperature, weighed again, and the weight was restored to its initial value with 70% ethanol. The sample solution was shaken thoroughly and filtered through 0.22-μm microporous filtration membranes. Two hundred microliters of the obtained filtrate was diluted to 1.0 mL with 70% ethanol to afford the test solution.

### Data analysis

MS data acquisition and processing were carried out by MassHunter Workstation software LC/MS Data Acquisition version B04.01 for 6460 Series Triple Quadrupole (Agilent Technologies, Santa Clara, CA, USA) and MassHunter Workstation software Quantitative Analysis version B 07.00/Build 7.0.57.0 for QQQ (Agilent Technologies, Santa Clara, CA, USA). SIMCA-P software (version 15.0.2, Umetrics, Umea, Sweden) and Heatmap Illustrator 1.0 software^35^ were employed for PCA, OPLS-DA and HCA, respectively. The thirty-five batches of *S. acutum* stem samples were divided into high- and low-content groups by comparing the average content of sinomenine according to OPLS-DA. HCA was performed on the logarithm of the contents of 11 alkaloids in the thirty-five batches of *S. acutum* stem samples. The cluster distance analysis and heat map were obtained by using the Euclidean distance.

## Supplementary information

Supplementary Information.

## Abbreviations

EIC: Extracted ion chromatogram
ESI: Electrospray ionization
HCA: Hierarchical cluster analysis
LLOQ: Lower limit of quantification
LOD: Limit of detection
MRM: Multiple reaction monitoring
PCA: Principal component analysis
RSD: Relative standard deviation
UHPLC-QQQ-MS/MS: Ultrahigh-performance liquid chromatography coupled with triple quadrupole tandem mass spectrometry
WADA: World Anti-Doping Agency
