# Supplementary material for: Discovery of chemical markers for improving the quality and safety control of Sinomenium acutum stem by the simultaneous determination of multiple alkaloids using UHPLC-QQQ-MS/MS
Source: Sci Rep. 2020 Aug 25;10:14182. doi: 10.1038/s41598-020-71133-4 (PMC7447749; doi:10.1038/s41598-020-71133-4)
Supplement: Supplementary file 1 — Supplementary Information. [file 41598_2020_71133_MOESM1_ESM.docx]

**Supplementary material**

**Discovery of chemical markers for improving the quality and safety control of *Sinomenium acutum* stem by the simultaneous determination of multiple** **alkaloids using UHPLC-QQQ-MS/MS**

Yu-Feng HUANG^1,2^, Fan HE^1^, Can-Jian WANG^1^, Ying XIE^1^, Yan-Yu ZHANG^1^, Zhen SANG^2^, Ping QIU^3^, Pei LUO^1^, Sheng-Yuan XIAO^4^, Jing LI^2^, Fei-Ci WU^3^, Liang LIU^1,5, *^, Hua ZHOU^1,5, *^

^1^ Faculty of Chinese Medicine and State Key Laboratory of Quality Research in Chinese Medicine, Macau University of Science and Technology, Taipa, Macao, P.R. China; ^2^ Institute of International Standardization of Traditional Chinese Medicine, Shanghai University of Traditional Chinese Medicine, Shanghai 201203, P.R. China; ^3^ Hunan Zhengqing Pharmaceutical Company Group Ltd. Huaihua City, 418000, P.R. China; ^4^ College of Chinese Medicinal Materials, Jilin Agricultural University, Changchun 130118, P.R. China; ^5^ Joint Laboratory for Translational Cancer Research of Chinese Medicine of the Ministry of Education of the People’s Republic of China, Macau University of Science and Technology, Taipa, Macao, P.R. China

* Correspondence to Hua ZHOU (hzhou@must.edu.mo, Tel: +853-88972458, Fax：+853-28825886) and Liang LIU (lliu@must.edu.mo, Tel: +853-8897 2238, Fax: +853-2882 7222)

**Method validation**

*Linearity*

The linearity of the method was evaluated by analyzing the standards at seven concentration levels in triplicate at each level. The calibration curves were constructed by plotting the peak area (*y*) of analytes versus concentrations (*x*) of analytes using a multiple regression linear regression model. The determination coefficient (*R^2^*) of the calibration curves should not be less than 0.998. The lower limit of quantification (LLOQ) is defined as the lowest concentration could be determined with a signal-to-noise ratio of 10:1. The limit of detection (LOD) is defined as the lowest amount of analyte that could be detected and determined as the concentration with a signal-to-noise ratio of 3:1.

*Precision*

The precision was measured at one concentration level of standard solution for six times continuously. The precision was defined as the relative standard deviation (RSD) of the measured peak areas. The RSD determined at each concentration level should not be larger than 5%.

*Repeatability*

The repeatability was determined by testing a sample which was separated into six portions and extracted separately, starting from the powdering to the end of UHPLC-QQQ-MS/MS analysis. The RSD of the content level of each compound should not be larger than 5%, after determining the concentrations of six copies.

*Stability*

The stability was determined by testing a test solution stored at room temperature repeatedly over 0, 2, 4, 6, 8 and 12 h in one day. The RSD of the peak area of each compound should not be larger than 5%.

*Recovery*

The recovery was determined by spiking a certain amount (approximately 0.25 g) of a sample of *S. acutum* stem with a known amount of the mixed standards repeatedly for six times (n = 6). The recovery was calculated by the equation below:

Recovery (%) = (total amount detected − original amount) / amount spiked × 100%

The recovery rates of the eleven compounds should be within the range from 95% to 105%, and the RSD of the recovery rate of each compound should not be larger than 5%.
